# HannibaL – the model curriculum at Hannover Medical School: targets, implementation and experiences

**DOI:** 10.3205/zma001265

**Published:** 2019-10-15

**Authors:** Volker Paulmann, Volkhard Fischer, Ingo Just

**Affiliations:** 1 Hannover Medical School, Hannover, Dean of Studies Office, Evaluation & Academic Controlling, Hannover, Germany; 2 Hannover Medical School, Hannover, Dean of Studies, Hannover, Germany; 3 Hannover Medical School, Institute for Toxicology, Hannover, Germany

**Keywords:** model curriculum, medical licensing regulations, curriculum development, faculty development, quality assurance, course evaluation

## Abstract

**Aim: **The model curriculum known as HannibaL is an integrated, professionally-based adaptive curriculum that began at the Hannover Medical School (MHH) during the 2005/06 academic year. HannibaL turns medical students into competent physicians through its patient-based interdisciplinary instruction. This paper provides an overview of the curriculum’s creation, educational content and philosophy and reflects on the experience that has been gathered. Also described are organizational and quality assurance measures which were also employed to implement the model curriculum.

**Method: **The central ideas and processes are reported in a primarily narrative manner in an attempt to present the information coherently. The aspects discussed are setting up the model curriculum, central features of teaching and exams with their underlying educational premises; organization and evaluation are also covered in the context of the research literature on curriculum and faculty development. Developing the teaching and learning culture of the model curriculum is also explored.

**Results: **The basic objectives were realized, including the design of learning spirals and intensifying the inclusion of patients and practical elements at the beginning of study. However, plans to allow students more freedom to pursue their own learning and research interests have not yet been satisfactorily implemented. Key areas to support teaching have been expanded (teacher training for instructors, student advising, course evaluations).

**Conclusion: **The model curriculum and its aims are widely recognized and supported not only by medical students and instructors, but also external committees and experts. As a consequence, HannibaL will be developed further in upcoming years to implement the objectives which have not yet been met and to master new challenges faced by medical education.

## Zusammenfassung

**Zielsetzung: **Zum Studienjahr 2005/06 wurde an der Medizinischen Hochschule Hannover der Modellstudiengang HannibaL (Hannoverscher integrierter berufsorientierter adaptiver Lehrplan) eingerichtet, der durch patientennahen, fachübergreifenden Unterricht die Studierenden zu kompetenten Ärztinnen und Ärzten ausbildet. Der Beitrag gibt einen Überblick über die Entstehung, die inhaltlichen und didaktischen Prämissen und reflektiert die gesammelten Erfahrungen. Ergänzend werden Maßnahmen der Studiengangsorganisation und der Qualitätssicherung beschrieben, die zur Umsetzung der Modellstudiengangsziele zum Einsatz kamen.

**Methodik:** Die zentralen Ideen und Prozesse werden überwiegend narrativ berichtet. Dabei wird eine kohärente Darstellung angestrebt. Die Einrichtung des Studiengangs, zentrale Merkmale der Lehre, der Prüfungen und ihre didaktische Prämissen, ihre Organisation und Evaluation werden ebenso wie die Entwicklung der Lehr- und Lernkultur im Modellstudiengang vor dem Hintergrund der Forschungsliteratur zur Curriculumsentwicklung und zum Faculty Development diskutiert.

**Ergebnisse:** Wesentliche Ziele – wie der Aufbau von Lernspiralen, die verstärkte Einbindung von Patienten und Praxiselementen von Beginn des Studiums an – konnten realisiert werden. Pläne, für die Studierenden vermehrt Freiräume für eigene Lern- und Forschungsneigungen zu schaffen, wurden dagegen noch nicht befriedigend umgesetzt. Schlüsselbereiche zur Unterstützung der Lehre (Didaktikkurse für Lehrende, Studierendenberatung, Lehrevaluation) wurden ausgebaut.

**Schlussfolgerung: **Der Studiengang und seine Ziele finden bei Studierenden und Lehrenden sowie bei externen Gremien und Gutachtern breite Anerkennung und Unterstützung. Auf dieser Grundlage soll in den kommenden Jahren die Weiterentwicklung von HannibaL erfolgen, um einerseits noch nicht realisierte Ziele umzusetzen und andererseits neuen Herausforderungen zu begegnen, die auf die medizinische Ausbildung zukommen.

## Introduction

In 1965, the Hannover Medical School (MHH) was founded as an independent university to anchor another medical school in Lower Saxony, Germany. As such, the MHH represents a relatively new voice in the ensemble of medical schools. During this time, the work on educational reform in teaching and the inclusion of students in decision-making processes have also had a significant tradition [1], [2]. In line with the new direction striven for during the university’s founding, at the turn of the millennium teachers and students jointly determined how the medical degree program at MHH should be newly structured. Described here are the most important ideas, processes and influential factors that have shaped MHH’s HannibaL curriculum over the past 13 years. Our approach is primarily narrative and includes examples that demonstrate the character of the model curriculum and its realization. Focus is also placed on insights regarding what has been achieved and what still needs to be addressed in light of the current research on curriculum and faculty development.

## 1. Setting up the model curriculum

In Germany, the last two decades of the twentieth century were shaped, from the point of view of medical educators, by the drafting of new “Regulations for the Licensing of Doctors” (ÄAppO), which were meant to form the basis for modern medical education [3], [4]. With the passage of these regulations in 2002, medical education became more practice-based and saw a stronger linking of preclinical and clinical content [https://www.gesetze-im-internet.de/_appro_2002/BJNR240500002.html]. With the model clause (§ 41), which had already been introduced in 1999, German medical schools had larger freedom overall to employ new curricular approaches. Under the leadership of the then Dean of Studies, Prof. Dr. Hermann Haller, a studies task force was created at MHH in 2004 to develop the ideas that had already been selectively implemented in the existing conventional curriculum. This included the division of the clinical years into three rotating blocks to enable the formation of smaller study groups and was first implemented in the 2002/03 winter semester. The inclusion of patients in teaching was pushed forward in response to the new medical licensing regulations, and the study program’s status as a model curriculum allowed the use of teaching hospitals to provide instruction that included patients. An important motive for further reform was the observation that, with up to 350 medical students each year and additional students in the preclinical phase, the structures anchoring the medical undergraduate education did not offer sufficient room to implement patient-centered, competency-based training [5]. In this case, the introduction of a model curriculum offered more leeway to cope with the high number of admissions. With the support of the Lower Saxon Ministries of Science and Social Affairs it was possible to develop curricular content that, after only one year of preparation, saw the enrollment of 270 students in the model curriculum for the 2005/06 winter semester. With the implementation of *HannibaL*, the conventional undergraduate program stopped enrolling new students and ended in 2009.

The model curriculum strives to produce competent physicians who are well-prepared to master the contemporary practice of medicine. This is achieved through

1. consistent linking of theoretical and clinical subjects and
2. interdisciplinary learning blocks.

This interdisciplinary approach pertains not only to the natural sciences and medical subjects, but also to the imparting of psychosocial skills within the clinical context so that students can adequately apply their acquired knowledge later when providing primary care [6]. The underlying concept of the physician is based on a critical understanding of scientific medicine and its relevance to medical practice, without basing it on a specific medical field or job in healthcare (doctor’s office, clinics, hospitals, university teaching hospitals). To foster this professional profile, classical interviews were introduced in 2006-2007 as part of the selection procedures followed by all German universities (*Auswahlverfahren der Hochschulen/AdH*). Three times the number of available university places within this quota to be admitted under the AdH quota are invited for classical interviews. Since up to 60% of admissions correspond to the number allowed by the universities’ selection procedures (AdH), the number of interviewees amounts to around 400 prospective students. The interviews, which are held by a team of two members of the teaching staff, provide an opportunity for applicants to present themselves and their activities in the areas of the arts and culture, science, athletics and social engagement. The pre-requisite for an invitation to such an interview is the selection of MHH as first preference.

## 2. Teaching and testing: educational premises

Three approaches were developed as educational premises for the model curriculum:

- Education of physicians using patients;
- Organization of teaching in terms of a learning spiral;
- Opportunity for students to take up a structured concentration during medical studies.

**Figure 1:**
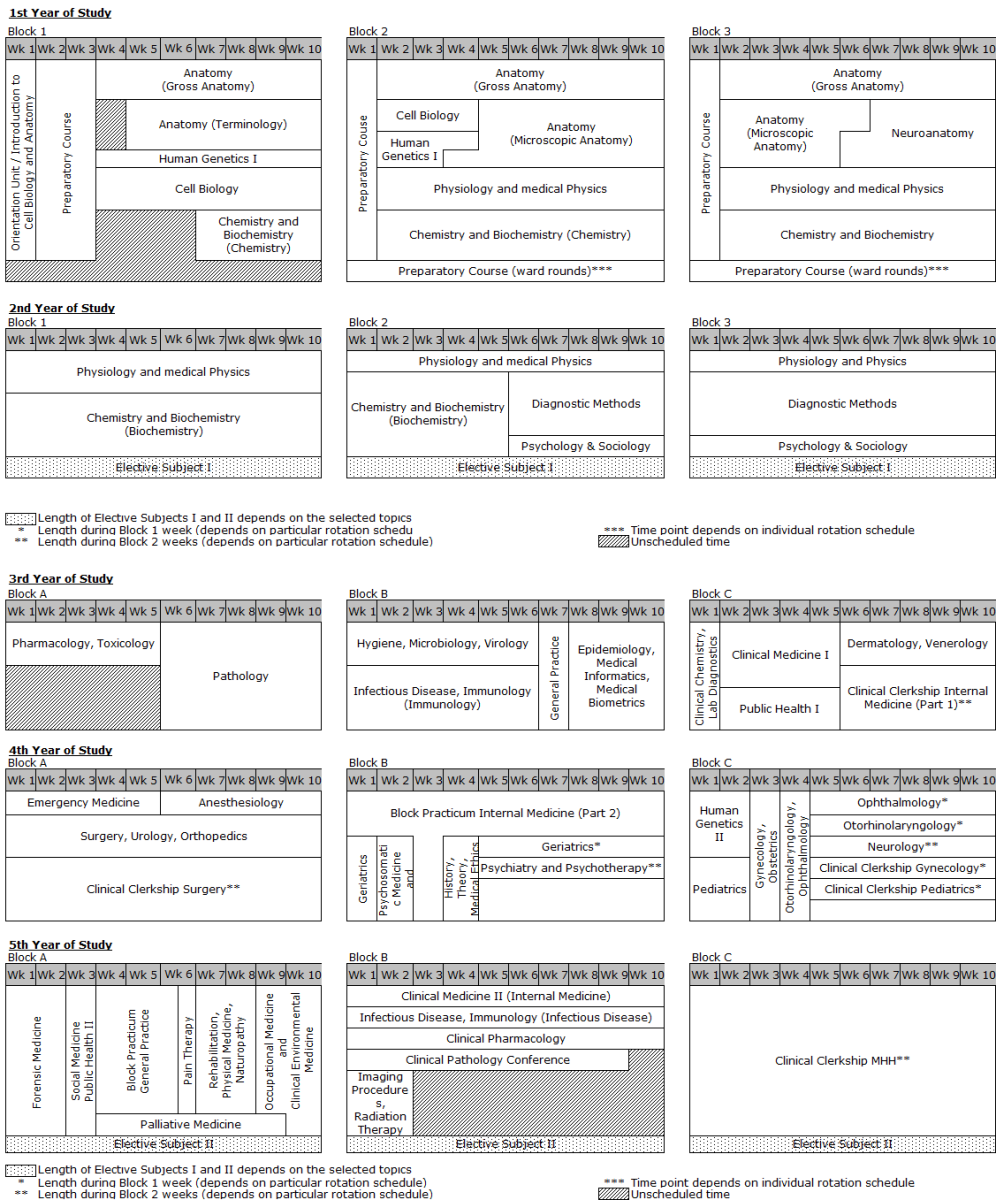
*HannibaL *model curriculum for the academic year 2018/19

The curriculum was designed to consist of modular courses based on the subjects listed in the German “Regulations of the Licensing of Doctors” (ÄAppO). Figure 1 (Fig. 1) shows the current course sequences for 2018/19.

As a result, the organization of the model curriculum retains its basic alignment with other medical schools and student transfers remain possible during undergraduate medical education. In addition, entirely new courses specific to the model curriculum have been introduced and, already in the first weeks of the study program, focus more strongly on patient-based teaching linking topics throughout the course of study. These include preparatory courses in the first academic year with the presentation of real patient cases and clinical rounds [7], the module on diagnostic methods using actors [8]; the final OSCE (*Objective Structured Clinical Examination*) in the second year of study; and the interdisciplinary preparatory course II for clinical medicine (later: Clinical Medicine I) in the third year; and the module covering differential diagnoses and therapy (later: Clinical Medicine II) in the fifth year of study. The teaching of internal medicine is an example of the development of such a longitudinal spiral and the strengthening of patient-based teaching in all years of study [5].

 Other examples can be seen in Rehabilitative Medicine [7] or Pharmacology, which is anchored in the first, third and fifth years of study.

Practical training in direct contact with patients is also enabled by visits to academic teaching hospitals, medical practices and rehabilitation clinics, as well as by block practicums and the final practice-oriented year at MHH or another of its teaching hospitals. The recurrent principal of repetition that can be seen here – but in another context – and sequential specialization correspond to the concept of the learning spiral [9]. At the same time, this learning and teaching strategy aims to form synergies to allow space and time for individual concentrations. High value was therefore placed on expanding e-learning at MHH parallel to the introduction of HannibaL. The learning management system ILIAS was augmented in 2006-2007 to provide support for teaching. All of the modules in the curriculum are represented on the e-learning platform based on the structure and organization of the study program. In addition, the university’s own *Medical Schoolbook* was introduced as an innovative, interactive learning environment with which case-based learning is encouraged (Cell Biology, Emergency Surgery, etc.). These different approaches primarily strive to help students in the self-study phases and with the learning spirals by providing the necessary resources.

At the module level, problem-based learning (PBL) is anchored in individual cases, for instance, in Pediatrics and General Practice. Despite all pedagogical efforts, the traditional lecture is still dominant in the model curriculum even though, in many instances, interactive elements have been incorporated into the teaching, such as the use of TED systems.

An open window of time is fixed in the fifth year of study that can be used to focus on a chosen topic. This is primarily intended for the medical dissertation. The structured doctoral degree program *StrucMed* [10], which has spaces for up to 65 students each year, has taken on the task of improving the quality of doctoral thesis in medicine. Alongside the doctoral degree, around 50 elective subjects offer the opportunity to concentrate on a chosen specialty.

The reorganization of the assessments and exams is an innovative feature of the model curriculum. Instead of the summative M1, the first part of the German state examination in medicine, the scores on the individual module assessments during the first phase of study are cumulative. These individual assessments test knowledge close to the time it is learned and are meant to bypass in multiple steps the hurdle of a single summative assessment after two full years of study [11]. So, as not to unnecessarily prolong the duration of study, missed or failed assessments can be retaken promptly. For the most part, the assessments are administered as multiple-choice exams, a process that involved new technical, legal and organizational aspects as a computerized testing system was introduced [12], [13], [14].

## 3. Course organization and evaluation

Implementation of the curricular content and educational premises of* HannibaL* required the corresponding organization at the teaching level. The Office of the Dean of Medical Studies was expanded to relieve pressure on the medical departments. A special person was appointed for each study year to advise on matters relating to the study program and exams, something that up to this time had not been part of the conventional study program. By doing this, it was possible to cover the increased effort involved with organization and advising, especially as more assessments were introduced once the rotating blocks were introduced. The switch to three rotating blocks means that the class cohorts starting the third year of study are divided into three rotating groups which pass through the modules by alternating every 10 weeks. As a consequence, the affected departments must hold an increased number of lectures, but the patient-based teaching in the hospital setting can be spread out more evenly over the year.

Alongside the administration of the study program, course evaluations and matters pertaining to the regulations governing student admissions have been bundled into a second focal area and more staff hired. The campus-wide course evaluations at MHH had already been realized in 2002. The concept was adapted for the model curriculum and the existing tools were supplemented [15], [16]. In addition to the course evaluations and questionnaires on study conditions [16], MHH has also conducted alumni surveys since 2010 [17]. In 2017, a comprehensive evaluation of all instructors took place for the first time [18]. In addition to providing direct feedback to instructors and for curricular development, these course evaluations serve yet another purpose since 2008-2009. As a supplement to the existing performance-based research budget, a second budget for teaching based on performance was introduced as an additional incentive to reward departments for particularly good teaching [19].

**Figure 2:**
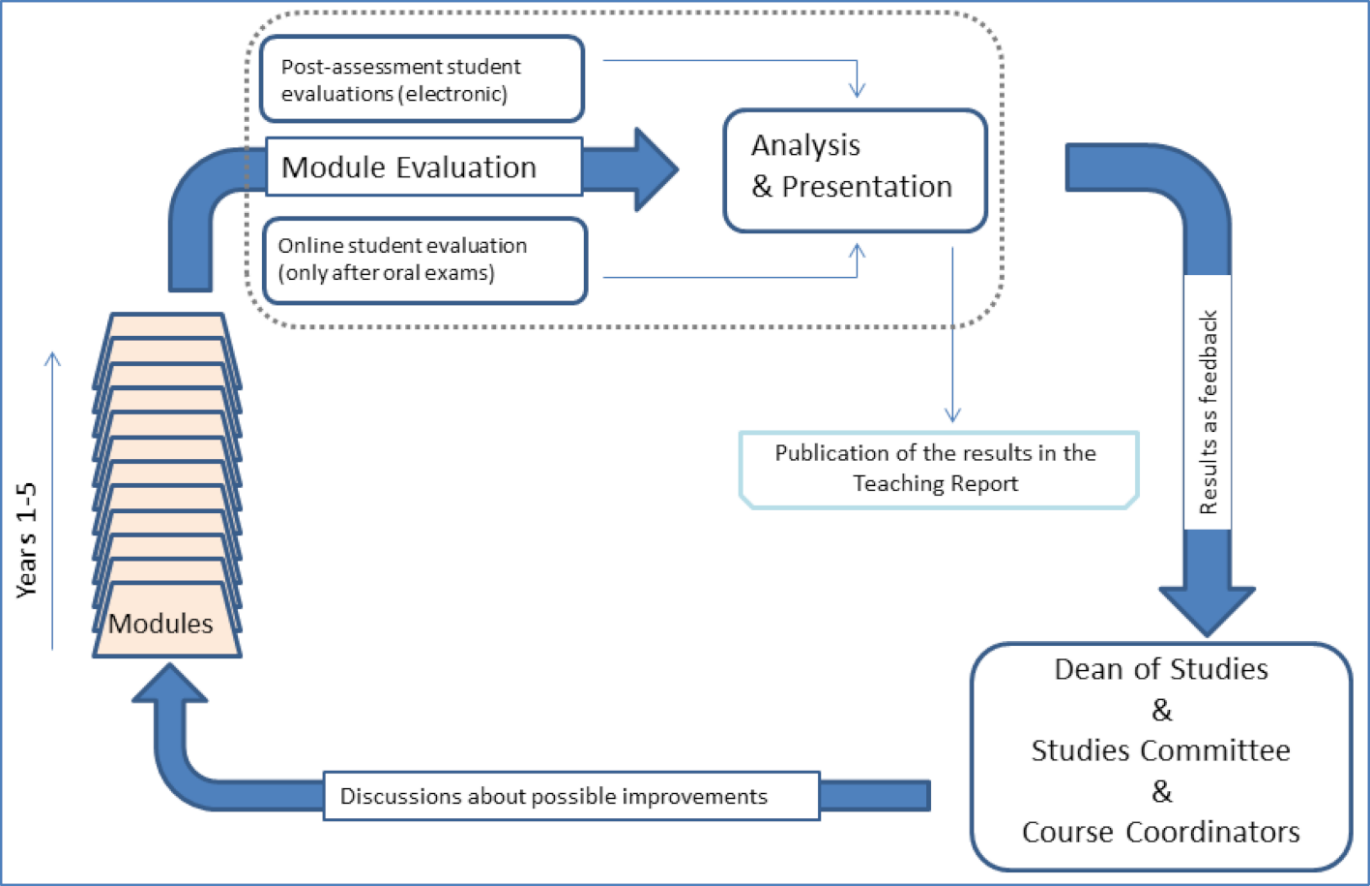
Course evaluations for the MHH model curriculum

On the level of academic self-administration the results of the Dean’s office efforts flow primarily into the work of the Studies Committee (see figure 2 (Fig. 2)) which functions as the central liaison in the communication between students and instructors at MHH.

The Studies Committee, composed equally of five teachers and five students, discusses and agrees upon all important curricular developments or prepares them for resolution by the MHH Senate; this body proved its value in the early phase of creating the model curriculum [19].

## 4. Developing the teaching and learning culture of the model curriculum

Communication and transparency are absolutely essential for the development of a new teaching and learning culture [20]. Different measures were implemented to hold discussions regarding changes to teaching on the broadest basis possible:

- Drafting of standardized module curricula with learning objectives and brief content descriptions [https://www.mh-hannover.de/hannibal.html];
- Internal publication of the module evaluations in the learning management system;
- Preparation of an annual teaching report, including module ranking and assessment statistics;
- Establishing performance-based funding for teaching.

**Figure 3:**
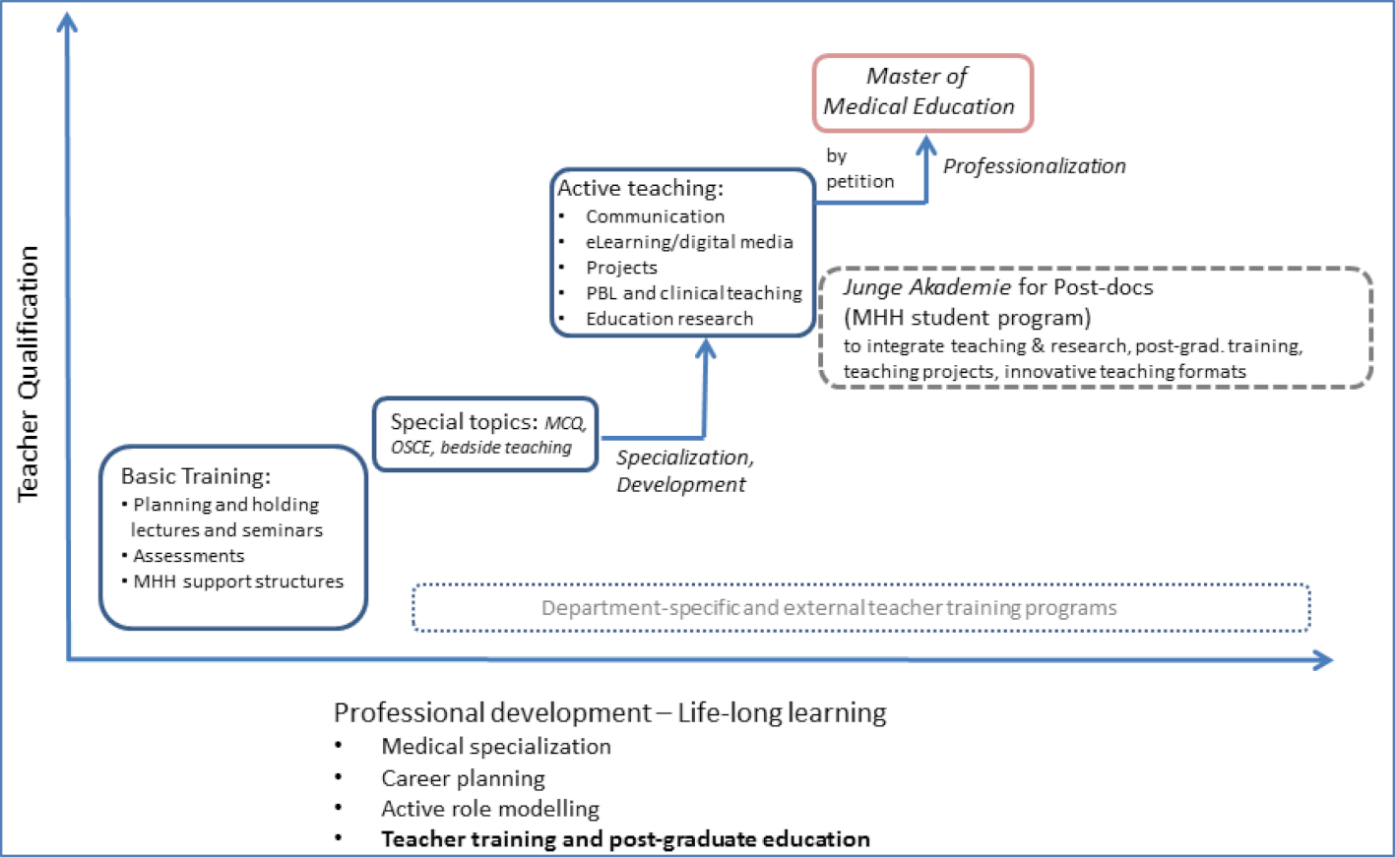
Promoting teacher qualification at MHH

To sustain a positive learning atmosphere lasting beyond the initial euphoria, different approaches for a faculty development program have been taken at MHH since 2005. The main one among these is the teacher qualification which is obtained in multiple steps (see figure 3 (Fig. 3)).

In addition to the basic teaching course (Basiskurs Lehre: 30 hours; required for qualification to teach at the university level) and the subsequent advanced training program (Aktiv in der Lehre: 200 hours), there is the option to participate in the post-graduate Master of Medical Education (MME) offered by the University of Heidelberg, the cap stone of MHH’s structures for professional development in teaching higher education. From the funds allotted to quality in academics, the MHH provides a grant each year to an instructor by assuming 50% of the tuition fees. The entire teacher training program at MHH was certified as the first program in Germany in 2016 by the *MedizinDidaktikNetz* of the *Medizinischer Fakultätentag*.

The motivation of individual instructors who are continually engaged in the process of improving teaching is reflected in the formation of a teacher network in 2012 (*Netzwerk Lehre)*. This network is a grassroots organization consisting of engaged and active instructors who exchange pertinent information, identify problems early and inform the university leadership, organize workshops on current issues in teaching, and have permanently established the annual teaching day (*Tag der Lehre*) at MHH.

## 5. Discussion, Summary, Outlook

The following summarizes the results by analyzing strengths and weaknesses and, for a better overview, presents them in a table (see table 1 (Tab. 1)). These aspects are discussed in detail in light of the literature to offer a basis to other medical schools and to further develop *HannibaL* while avoiding undesirable developments. This discussion follows the same sequence of topics as in the results section.

### 5.1. Setting up the model curriculum

In hindsight, it is clear that, as a result of the relatively short preparation time, the structural anchoring was not always able to keep up, particularly in the beginning phase, with the tempo of the curricular developments – even though sufficient time is recommended for the implementation phase [21]. Ad-hoc management with too few staff members was the consequence. In the following years this imbalance was righted through the use of tuition fees and, as of 2014, with its equivalent funding to improve the quality of academic study through the use of module coordinators, mentors for the Skills Lab, and the creation of new positions in the Office of the Dean of Studies. Also, the focus of core HannibaL modules was initially set and designed by the subject representatives (from internal medicine, biochemistry, pharmacology, and rehabilitative medicine) on the planning committee for the model curriculum. In individual instances, interdisciplinary workshops were held to organize the process of coordination among the different clinics and subject representatives (internal medicine, biometrics/medical informatics/epidemiology). The study program rules and regulations and the model curriculum (see sections 3 & 4) also underwent continual adaptation in the past years. The Studies Committee is often called upon to present convincing arguments, primarily with an eye on reducing the course volume. Progress was seen here only gradually since the subject representatives usually tended toward giving their own subjects higher value within the curriculum. The question of how to revise the curriculum with the active participation of all faculty members is one of the greatest challenges of change management [22], [23]. Looking back, the early formation and sustained involvement of an external advisory body, which was put into place in 2013, could have proved to be a corrective and certainly helpful measure.

#### 5.2. Content and educational approach of the model curriculum

In general, the structure of the model curriculum has proved valuable. The elimination of the hurdle posed by the M1 has gotten rid of the delays caused by missed or failed exams because it is now possible to compensate for such situations [11]. Above all, the integration of clinical and patient-based teaching in the first year of study has been an important aspect in the past 10-15 years. Accordingly, in the first two years of study more modules have been able to link themselves with clinical content and thus put a central strategy of the medical licensing regulations (§2, subs. 2 ÄAppO) into practice than would have been possible in a conventional study program. Cooperative efforts with academic teaching hospitals have contributed to accomplishing this and such collaborative relationships have intensified with the introduction of the model curriculum.

However, not all of the goals have been met equally. It has been seen that setting up networked and integrated courses requires a great amount of time and resources, as reported by other medical schools [24]. Creating additional coordinating positions to handle these increased efforts involved with organization and communication has been helpful in attaining goals. In terms of further curriculum development, MHH has already compared HannibaL against the learning objectives contained in the NKLM (National Competency-based Catalogue of Learning Objectives in Undergraduate Medical Education) and identified important approaches for integrating content and expanding competency-based teaching [25], [26].

Integration should also serve to create space and time for students to pursue individual academic interests. It can be stated that the largest discrepancy in the model curriculum is between what is needed to accomplish this and the actual situation. The student course load has increased markedly in comparison to 2002; the proportion of classroom-based teaching and courses with required attendance often exceed what is desirable from a pedagogical standpoint. This also negatively affects scientific training, another core aspect of *HannibaL*. It is true that the basics of science are taught to students primarily during the doctoral phase, and while MHH does have an above-average graduation rate, explicit approaches and instructions regarding this type of scientific research and writing have up to now only been weakly anchored in the curriculum. In response to this, a new longitudinal science module early on in medical study is being designed to impart scientific tools for medical practice. This encompasses lectures, courses and concludes with a research paper [27].

#### 5.3. Quality assurance: organization, structural anchoring and evaluations

Expanding the Office of the Dean of Studies created the framework for the organizational restructuring of the model curriculum. Centralizing the administration of exams and course scheduling relieved the academic departments so that capacities opened up. However, to ensure properly functioning processes, such as those pertaining to quality assurance, binding rules and requirements are necessary and which are often perceived of as burdensome bureaucracy. Appointing one person as the responsible coordinator for each module has proven to be valuable and facilitates communication about the structure and content of the model curriculum.

The comprehensive course evaluations are accepted by most instructors and students. The fact that a few modules consistently appear at the bottom of the student rankings demonstrates the limited influence of the existing feedback loop. More weight must be placed in the future on the didactical and organizational advising of the responsible course coordinators. Controversies also arise repeatedly regarding the performance-based allocation of funding for teaching. Finding an approach that transparently considers all module test scores, course evaluations and the investment of time while also creating incentives for good teaching remains a challenge. There was a second attempt after about 10 years to consider new incentives. Overall, the use of the established tools represents a strength of the model curriculum and these have been issued regularly by way of external assessment by the advisory committee and the central evaluation and accreditation agency (Zentrale* Evaluations- und Akkreditierungsagentur/ZEvA*) [28], [29].

#### 5.4. Teaching and learning culture, Assessments

Although basic satisfaction with the courses and teaching is evident in all student evaluations, the situation regarding assessments is worthy of discussion. The fact that in the first two years of study each module assessment is equivalent to a section of the M1 (see section 2) combined with the graded course content in years 3-5 lead students to regard most learning material as exam relevant. This can only be avoided by more strongly bundling assessments or returning to ungraded academic courses. In addition, the proportion of multiple-choice exams is too high for a model curriculum and there is too little focus on physician competencies so that in the coming years additional efforts will be needed regarding curriculum development. This notwithstanding, the integration of assessments into medical education poses a major challenge for medical schools [30].

From the perspective of instructors, a lack of time remains the main obstacle to greater commitment to teaching. Most of all, hospital teaching is enormously demanding in the face of increased cost pressures. As long as teaching is regarded as secondary to clinical practice and research, the teaching culture will remain mostly dependent on the personal dedication of individual people. The most important structure-based strategies over the medium term could be to include teaching responsibilities as a formal part of physicians’ hospital work schedules. Until this can be done, the only management tool to promote teaching efforts remains in the form of money and the budget and performance-based allocation.

#### 5.5. Evaluation of the model curriculum

Alumni surveys have been conducted at MHH since 2010 to further develop the model curriculum. Overall satisfaction with the study program among those surveyed has risen continually in past years from 2.6 to 2.1 on a five-point scale (1=very satisfied <> 5=very dissatisfied). Approximately 95% of MHH graduates take up employment in healthcare after completing their medical studies [17], [31]. The currently available options to empirically analyze the added value of *HannibaL* will nonetheless hardly silence all critics. This must not be seen as a reason to abandon science-based evaluation of study programs. On the contrary, even more initiative is desirable to verify the quality of education by comparing perspectives using a mix of methods, since medical education has become incredibly diverse over the past decade. Alongside model curricula and reformed or conventional study programs, yet other educational programs have been created outside of the state universities; among these are collaborations with German hospital operators and foreign universities. In light of this, science-based mentoring in medical education remains a critical issue. However, since the internal perspective of a university is by definition limited, proposals from the German Council of Science and Humanities (*Wissenschaftsrat*) can be seriously considered in the future when they involve the development of cross-university evaluation criteria by an expert group [32]. This could as a consequence sharpen the eye mainly for differences in focus between model and conventional curricula. Earlier studies have already shown that the quality of results depends on a number of factors, including those that do not fall within the influence of curriculum designers [33], [34], [35]. These limiting factors include the general legal provisions regarding student selection, increased economizing in the healthcare system, and the much discussed need for more physicians. The call to increase the number of university places at medical schools comes not only from policy makers and professional associations, but is also supported in part at MHH. Such support comes mostly from the clinical and surgical fields which are most strongly experiencing the scarcity of medical personnel. The friction between the administrative forces in the hospital that above all strive for cost reduction and the educational need to improve the academic quality often make the goals of the model curriculum appear a luxury.

#### 5.6. Conclusion and Outlook

The model curriculum embodied in HannibaL at MHH is unique in both content and program structure, even if not all goals have yet been reached. A reduction of courses with required attendance and strengthening scholarly competencies remain important challenges. The elimination of the M1 at MHH has not led to a decrease in theoretical knowledge since separate assessments take on the function of testing for knowledge. Instead, the elimination of a graded exam (in the form of the M1) has shown that learning difficulties at the start of study can be compensated for individually without losing time in the overall course of study. Early contact with patients in the first year of study and the related reflections on the role of the physician help, as do the interdisciplinary and longitudinally anchored modules, to link basic theory with clinical issues. The declared goal of preparing competent physicians for demanding jobs in healthcare without giving up on the proven standards in medical education is well on its way to being attained. Accordingly, the German ministries and the external advisory body unanimously recommended the continuation of the model curriculum in 2013. HannibaL also receives wide support from students and instructors at MHH. Continuing development and with it the opportunity to try out and implement new ideas is particularly viewed as a great advantage. Fostering and maintaining this positive atmosphere when dealing with upcoming changes, namely the application of the competency-based learning objectives (implementation of the NKLM, Master Plan Medizinstudium 2020) and the digitalization of medicine, is an important task (see table 1 (Tab. 1)).

## Competing interests

The authors declare that they have no competing interests. 

## Figures and Tables

**Table 1:**
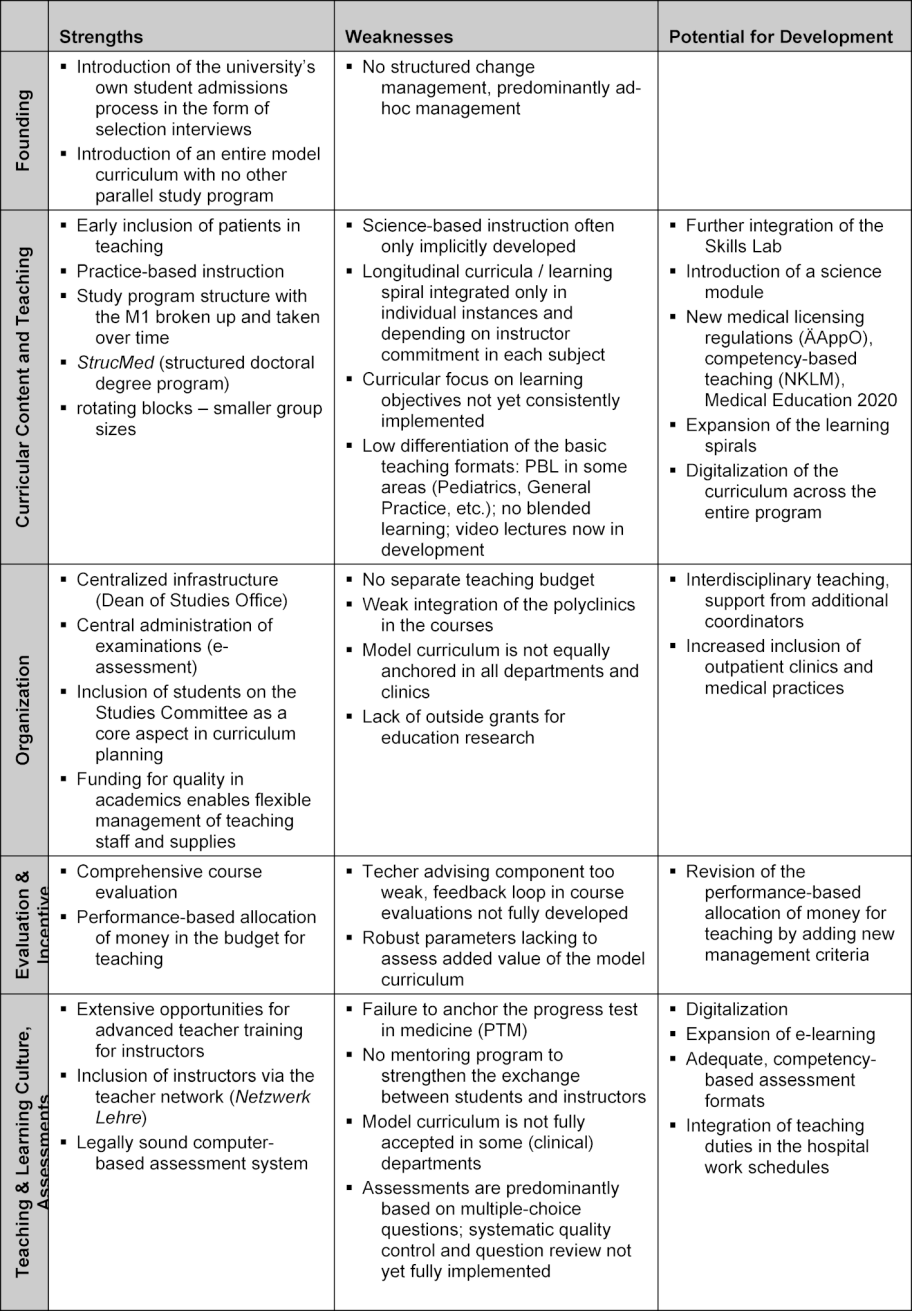
Strengths/weaknesses of the HannibaL model curriculum
